# COVID-19 Biomarkers for Critically Ill Patients: A Compendium for the Physician

**DOI:** 10.3390/neurolint15030056

**Published:** 2023-07-23

**Authors:** Federica Arturi, Gabriele Melegari, Antonio Giansante, Enrico Giuliani, Elisabetta Bertellini, Alberto Barbieri

**Affiliations:** 1 School of Anaesthesia and Intensive Care of University of Modena and Reggio Emilia, Via del Pozzo 71, 41125 Modena, Italy; 2 Anesthesia and Intensive Care, Azienda Ospedaliero Universitaria di Modena, Via del Pozzo 71, 41125 Modena, Italy; 3 School of Medicine, University of Modena and Reggio Emilia, Via del Pozzo 71, 41215 Modena, Italy; 4 Neuronguard, Neuron Guard Ltd., Windsor House, Station Court, Station Road Great Shelford, Cambridge CB22 5NE, 7170 Cambridgeshire, UK

**Keywords:** COVID-19 biomarkers, IL-6, endothelium dysfunction

## Abstract

Background: SARS-CoV-2 clinical manifestation and progression are variable and unpredictable, hence the importance of considering biomarkers in clinical practice that can be useful for both diagnosis and prognostic evaluation. This review aims to summarize, for intensive care physicians, the most recent state of knowledge regarding known COVID-19 in critical patients. We searched PubMed^®^ using the Boolean operators and identified all results on the PubMed^®^ database of all studies regarding COVID-19 biomarkers. We selected studies regarding endothelium, cytokines, bacterial infection, coagulation, and cardiovascular biomarkers. Methods: We divided the results into four essential paragraphs: “Cytokine storm”, “Endothelium dysfunction and coagulation biomarkers in COVID-19”, “Biomarker of sepsis”, and Cardiovascular lung and new perspectives. Results: The assessments of the severe COVID-19 prognosis should monitor, over time, IL-6, soluble Von Willebrand factor (VWF), P-selectin, sCD40L, thrombomodulin, VCAM-1, endothelin- Troponin, D-dimer, LDH, CRP, and procalcitonin. Metabolomic alterations and ACE2 receptors represent new perspectives. Discussion and Conclusions: Early identification of critically ill patients has been crucial in the first COVID-19 pandemic wave for the sustainability of the healthcare emergency system and clinical management. Only through the early identification of the most severe patients can they be provided with the most appropriate treatments.

## 1. Introduction

From the very beginning, the infection caused by SARS-CoV-2 has significantly impacted healthcare worldwide [1]. Its clinical manifestation and progression are variable and unpredictable, hence the importance of considering biomarkers in clinical practice that can be useful for both diagnosis and prognostic evaluation, especially for a better allocation of healthcare resources, as happened in different disasters or mass gathering events [2,3,4,5]. A characteristic of this virus is that it leads to a “cytokine storm” in the host induced by a hyperactivation of the immune response. This results in the engagement of various immunological mediators, such as chemokines, interferons, and interleukins; as a consequence, these can induce endothelium damage and endotheliitis. The endothelium damage is crucial in the severe clinical presentation of COVID-19: the pulmonary damage known as ARDS (acute respiratory distress syndrome) or the neurological injuries [6]. Patients with these severe clinical complications usually present modified plasmatic levels or alterations in coagulation parameters, thrombocytopenia, increased D-dimer, prolonged prothrombin time, or higher inflammatory protein levels such as C-reactive protein (CRP) and procalcitonin [7]. This review aims to summarize, for intensive care physicians, the recent knowledge regarding known biomarkers for COVID-19 infection to identify predictors of the most severe critical patients. To better understand the possible biomarkers of COVID-19 infection, it is essential to understand the pathophysiological determinants of its severity: cytokine storm, markers of endothelium damage, alterations in coagulation parameters, and biomarkers of sepsis.

### Pathophysiological Determinants of Severe Forms of COVID-19 Infection

In the first phases of infection, the virus enters target cells, nasal and bronchial cells, and pneumocytes by binding its spike (S) protein and the angiotensin-converting enzyme 2 (ACE2). At the same time, the protein TMPRSS2 promotes S protein activation and, therefore, the entrance of the virus into target cells. In addition, SARS-CoV-2, as with other respiratory viruses, causes the death of T lymphocyte cells and, as a consequence, an important lymphopenia. As the infection progresses, with additional viral replication, the integrity of the endothelial barrier is damaged, and this promotes a wide influx of immune cells, in particular neutrophils and monocytes, a key element leading to endothelitis [8]. The binding of SARS-CoV-2 to the ACE2 receptor reduces the local activity of ACE2, with a consequent increase in Angiotensin 2. Subsequently, the renin-angiotensin (RAS) system is activated, mediated, and induced by the involvement of the ACE2 receptor directly; this type of receptor has a strong expression in the lung endothelium. The hormonal cascade of RAS begins with the cleavage of the circulating, liver-derived angiotensinogen (AGT) by the protease renin (REN). This process leads to the formation of the decapeptide angiotensin I (ANG I). ANG I is later cleaved by the matrix metallopeptidase angiotensin-converting enzyme (ACE) [9,10,11]. ANG I is then cleaved by the matrix metallopeptidase angiotensin-converting enzyme (ACE). The product of this process is the peptide hormone angiotensin II (ANG II), the main RAS effector. ANG II activates many signal transduction pathways and promotes vasoconstriction, as well as hypoxic, oxidative, inflammatory, and proliferative events. Anyway, the activities of ANGII are counterbalanced by the activation of the ACE2/ANG1-7/MasR axis, with an opposite role that is, respectively, controlled by the angiotensin-converting enzyme 2 (ACE2). This process can induce leakage of pulmonary blood vessels through the stimulation of type 1 Angiotensin 2 receptors, which represent an important key point in the pathogenesis of ARDS. The increase in capillary leakage can also promote the onset of viremia, or the presence of the virus in the blood stream [12]. Furthermore, the over-activation of ACE/ANGII/AT1R signaling can also favor local inflammation and the “cytokine storm”. This cytokine cascade modulates the activities of pulmonary macrophages, dendritic cells, and neutrophils [10]. Macrophages containing the virus and expressing ACE2 produce elevated levels of IL-6; this cytokinin peak is responsible for the excessive inflammation typical of the disease and explains the increase in CRP levels. Blood levels of pro-inflammatory cytokines tend to be higher as disease severity progresses. This rise is also associated with the depletion of T cells. Indeed, the typical “cytokine storm” caused by the overactivation of the inflammatory response, which is observed especially in more severe patients, can lead to a negative alteration and response of the immune system and a minor response to medical treatments. Moreover, high levels of IL-6 have been related to the Macrophage Activation Syndrome, which consists of an important rise in macrophage levels and is responsible for lung inflammation and damage [13]. Furthermore, the excessive production of pro-inflammatory cytokines has been identified as a significant cause of coagulation disorders. The immune activation induces endothelial damage on the vascular surface and on macrophages caused by the production of tissue factors and phosphatidylserine; this process activates the coagulation cascade. Endothelial cells become procoagulant through the disruption of the glycocalyx and loss of anticoagulant proteins [14]. Lastly, the tropism of SARS-CoV-2 for the ACE2 receptor expressed by endothelial cells is one of the crucial points to explain the severity and systemic damages induced by Novel Corona Virus 19. The mechanism by which the virus infects endothelial cells leads to cellular dysfunction and apoptosis and, consequently, to inhibition of the normal antithrombotic activity of the endothelium. In addition, the endothelial cells are activated by pro-inflammatory cytokines, and then they promote the release of Von Willebrand factor (VWF). This process increases the interaction between platelets and vessel walls and leads to thrombotic microangiopathy [15]. Endothelium dysfunction also determines an alteration of vascular equilibrium, with more vasoconstriction and, consequently, organ ischemia, inflammation, and tissue edema [16]. These observations suggest that the main pathogenetic mechanism of this type of viral infection could be endothelial cell damage and the subsequent induction of endotheliitis.

## 2. Materials and Methods

**Figure 1:**
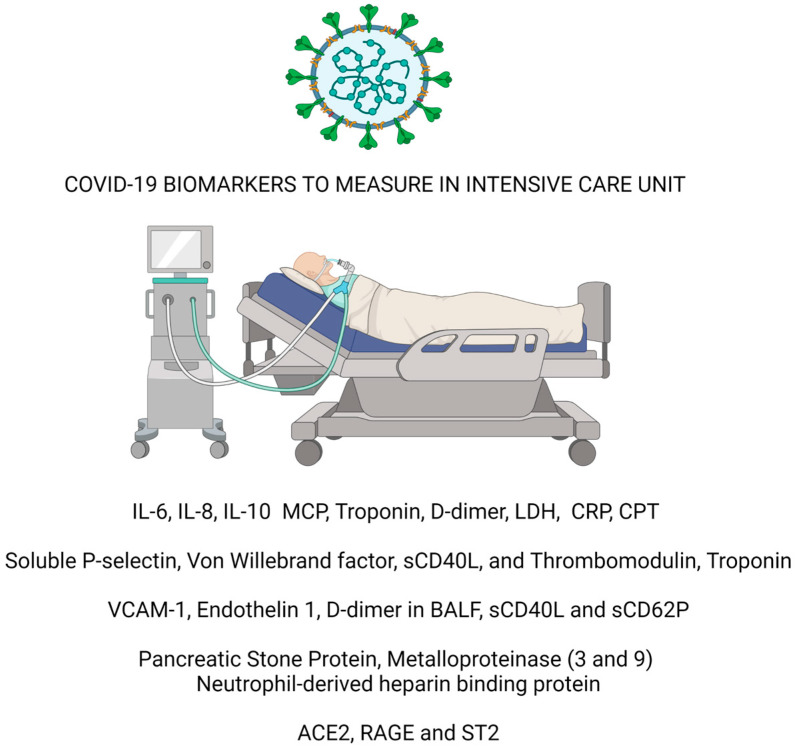
Summarizes the principal findings of the review.

This review follows the 2019 version of the scale for the quality assessment of narrative review articles, “SANRA” [17]. We searched PubMed^®^ for the past three years, using the Boolean operators AND, OR, and NOT. Inclusion criteria: we identified all results on the PubMed^®^ database of all studies regarding COVID-19 biomarkers. We selected studies using Boolean operators’ endothelium, cytokines, bacterial infection, and coagulation biomarker. We divided the results into 4 essential paragraphs: “Cytokine storm”, “Endothelium dysfunction and coagulation biomarkers in COVID-19”, “Biomarker of sepsis”, and Cardiovascular, lung and new perspective biomarker” resumed in Figure 1.

### 2.1. Cytokine Storm

Cytokine dysregulation during COVID-19 infection is one of the mechanisms contributing to disease severity; indeed, the efficacy of immunomodulatory therapies has been investigated in multiple clinical trials. High cytokine levels in patients with respiratory failure, acute respiratory distress syndrome, and hyperinflammation are common features of SARS-CoV infection. For that reason, the medical community focused its attention on Interleukins (IL) involved in the pathophysiology of severe forms of COVID-19 infection: IL-8, IL-10, and IL-6. There are several changes in cytokine levels in COVID-19 patients during their length of stay: Jøntvedt Jørgensen et al. demonstrated in their study that IL-6 combined with monocyte chemotactic protein (MCP) were both high in patients with respiratory failure; these proteins had a significant inverse correlation with the Horowitz index ratio and showed the most significant area under the curve in ROC analyses [18]. MCP is expressed by monocytes and is responsible for their chemotaxis and activation; it is stimulated by several cytokines and plays a fundamental role in the inflammation phases of COVID-19. The impact of circulating monocyte cells and inflammatory cytokines in the context of COVID-19 clinical progression was investigated by Pirabe et al. [19]. In their research, MCP levels in COVID-19 patients increased in older patients. Still, at the same time, the fact that the highest MCP levels have been found in elderly patients with adverse outcomes suggests that an unfavorable outcome could be a possible consequence of an increase in MCP. Interestingly, the authors also demonstrated the importance of monitoring IL-6 plasma levels. This interleukin did not vary by the outcome at the beginning of the clinical presentation; anyway, patients with a less severe course of the infection showed a rapid decline of IL-6 over time. Indeed, persistently elevated levels have been demonstrated in non-survivors [19]. These results are from Santa Cruz et al., who divided IL-6 levels according to disease stages and demonstrated that increasing levels of IL-6 are associated with the severity of disease, so as to identify patients more likely to evolve toward more severe stages of COVID-19 [20]. Furthermore, Espíndola et al. evaluated the levels of cytokines in the cerebrospinal fluid (CSF) of patients with COVID-19, observing different CSF and serum patterns of cytokines according to the neurological clinical conditions. Parainfectious cerebral neurologic system inflammatory syndromes related to COVID-19 infection were associated with elevated levels of IL-6 in CSF rather than the elevated plasmatic levels recorded and measured in encephalopathy [21]. In the end, Van Singer et al. investigated and demonstrated a predictive value of IL-16 in patients with COVID-19 when measured at presentation to the emergency department. In particular, this marker has good predictive accuracy for 30-day clinical deterioration and oxygen requirement [22]. At the same time, this research group focused on the TREM-1 receptor, which would be the triggering receptor expressed in myeloid cells and found on the surface of neutrophils circulating in the blood stream and of mature monocytes/macrophages. They hypothesized that the measurement of this receptor could have the best predictive accuracy for day-30 intubation/death. It has been demonstrated the value of repeated measures of IL-6 in critically ill COVID-19 patients, identifying patients with a high risk of poor prognosis [23]. The serum concentration of IL-6 could also be useful to monitor the clinical response to pharmacological medical treatments in severe COVID-19 patients, as observed by several authors [24,25,26]. Furthermore, according to Schultheiß et al., the monitoring of the IL-6 trend is useful to predict long-term sequelae; these data are in accordance with the research of Queiroz et al. [27,28]. In addition, IL-6 was not the only cytokine investigated in severe COVID-19 patients; recently, Melero et al. focused their attention on the cellular expression of IL-8 related to neutrophil modulation and the chemotaxis process [29]. Furthermore, interesting results were also detected by Bain et al. and Guasp et al., confirming the role of IL-8 and IL-10 in cases of ARDS or encephalopathy related to COVID-19 [30,31]. IL-10 has shown some interesting evidence as a prognostic factor, according to Han et al. [32]. The topic of cytokine concentration is fundamental in the COVID-19 assessment, as remarked by Del Valle et al. Indeed, this receptor is strongly involved in endothelium dysfunction, which is an essential key point to understanding the pathophysiology of COVID-19 [22]. Results from trials and prospective studies are summarized in Table 1.

### 2.2. Endothelium Dysfunction and Coagulation Biomarkers in COVID-19

The implemented vascular permeability and coagulopathy in COVID-19 infection are due to endothelium dysfunction, so the early identification of endotheliopathy has been investigated to find a predictive value and therapeutic target. Goshua et al. investigated in the first pandemic wave of COVID-19 the role of the following endothelial biomarkers: soluble P-selectin, and coagulation biomarkers such as Von Willebrand factor (VWF), sCD40L, and soluble thrombomodulin [33]. In their research, endothelial cell and platelet activation biomarkers were significantly elevated in intensive care unit (ICU) patients compared with non-ICU patients, including VWF. Vieceli Dalla Sega et al. found significantly high levels in patients with severe forms of COVID-19 of endothelial biomarkers such as VCAM-1, endothelin-1, and thrombomodulin. In particular, endothelin-1 remained stable in nonsurvivors but increased over time in survivors [34]. The endothelium’s dysfunction also has a direct effect on coagulation alterations, which are more evident in patients with COVID-19 infection. Al-Samkari et al. showed in their study a higher rate of thrombotic events in these patients, especially in several venous districts but also in arterial ones. In some patients, it was also observed that there was a clotting of the circuit during CVVH therapy, with the necessity of an increase in the heparin dose. The risk of these complications is directly associated with alterations in coagulation parameters, such as an increase in D-dimer. On the contrary, other patients show an increased risk of bleeding complications associated with thrombocytopenia; for this reason, the role of a potential intensification of anticoagulant therapy is uncertain [35]. Furthermore, Nossent et al. underlined a greater increase of coagulation markers, such as thrombin-antithrombin complexes and D-dimer, in bronchoalveolar lavage fluid (BALF) than in blood. This aspect confirms a major activation of coagulation in the pulmonary system, promoting a direct bronchial coagulopathy. This evidence does not exclude the activation of systemic coagulation and thrombotic events in these patients [36]. On the other hand, Hamzeh-Cognasse et al. hypothesize the involvement of platelets in the thromboinflammation induced by the Novel Coronavirus 19. They studied the roles of two proteins produced by platelets, sCD40L and sCD62P. In particular, patients with COVID-19 infection presented elevated levels of these proteins compared to other ICU patients [37]. The involvement of endothelium is joint in both respiratory manifestations and neurological and vascular manifestations, favoring the diffusion of microthrombi and altering vascular permeability [38,39]. Differently, Pine et al. investigated markers of angiogenesis, finding that angiopoietin-2, follistatin, and plasminogen activator inhibitor-1 (PAI-1) were prognostic and predictive of in-hospital mortality [40]. The authors demonstrate that the non-critical and critical phases of COVID-19 disease may be driven by distinct mechanisms involving endothelial cell function. In this context, the angiopoietin protein is an interesting biomarker; indeed, since the first measurements, it could predict the severity of the disease, but it could also be a marker of therapy [41]. The advantage of plasma measurement of angiopoietin to predict ICU admission in COVID-19 patients is still known from the first experience of Smadja et al. Anyway, the first trial involving the angiopoietin protein as a molecular target did not show any clinical improvement [42]. The Angiopoietin protein is not only an endothelium biomarker, but the proteins of the Selectin family play an interesting role as early mediators of the adhesion of activated polymorphonucleates to endothelial cells in inflammatory states. These proteins have been found and described in severe respiratory COVID-19 patients [43,44]. Since the first thrombotic complications in infected patients, an alteration of coagulation and hemostasis processes was evident; for this reason, the coagulation was an object of speculative interest. The meta-analysis of Adrianto et al. confirmed the VWF role in COVID-19 prognosis; on the other hand, Yong Li et al. demonstrated the dynamic relationship between D-dimer levels and COVID-19 severity [45,46]. Differently, among all endothelium biomarkers, the measurement of D-dimer is more common in clinical practice, which makes it a more feasible and useful marker. Von Willebrand factor (vWF) and coagulation screening tests (PT and a PTT), antithrombin (AT) III, clotting factor VIII, fibrinogen, and D-dimer in COVID-19 patients are a useful bundle of tests that are easy to perform daily. Indeed, when they are combined, they show a good correlation with the prognosis [47]. Results from trials and prospective studies are summarized in Table 2.

### 2.3. Biomarker of Sepsis

It has been demonstrated that there is a common feature between SARS-CoV-2 infection and bacterial sepsis. Indeed, in both, we can notice an important lymphopenia involving all cell populations (B, T CD4+, T CD8+) and a decrease in mHLA-DR levels. In addition, we can observe an increase in cytokine levels, both immunosuppressive (IL-10) and inflammatory ones (IL-6), and a rise in plasma IFNα2 concentrations. As observed in bacterial sepsis, the onset of ARDS can amplify immune alterations induced by COVID-19 through direct cytotoxic action and inhibition of the anti-inflammatory response [48]. The consequence is the induction of an immunosuppressed status in COVID-19 patients that develops ARDS, promoting the severity of the disease and increasing the risk of mortality. A protein that could have a central role in COVID-19 bacterial sepsis is Heparin Binding Protein (HBP). It is produced by neutrophils in the first phases of infections before the occurrence of the organ dysfunction typical of sepsis. It is also able to induce endothelial dysfunction and lung and kidney damage. Mellhammar et al. demonstrated high HBP levels in COVID-19 patients who then developed organ insufficiency, assuming the protein’s role in predicting the onset of organ failure in patients with severe forms of infection [49]. Differently, procalcitonin (PCT) is a protein released in tissues during inflammation; it can indicate a condition of hyperinflammation in the presence or absence of a bacterial infection in critically ill patients. Serum levels of PCT have been supposed to be correlated with the severity of COVID-19 infection, according to Voiriot et al. [50]. Usually, PCT is a biomarker specific for bacterial infection; for that reason, it could have a double meaning: first of all, monitoring COVID-19 prognosis, and at the same time, monitoring bacterial infection secondary to SARS-CoV-2 pneumonia, a common clinical complication of the infection. A biomarker of sepsis received a lot of interest in the medical community during the pandemic wave, but the results are controversial. According to Smilowitz et al., the measurement of CRP in COVID-19 patients is useful as an approach for risk stratification for COVID-19 patients. Differently, Pink et al. described that PCT and CRP may be helpful in early identifying secondary bacterial infections and guiding the use of antibiotic therapy [51,52]. The role of C reactive protein is still controversial in the case of sepsis; indeed, the measurement lacks sensitivity or specificity, but the prognostic value increased even if combined [53,54]. Also, PCT has controversial results as a COVID-19 biomarker, as underlined by Carbonell et al., even though they have confirmed the potential role of this biomarker in cases of bacterial infection or sepsis [55]. Recently, the measurement of Pancreatic stone protein showed a possible prognostic role, especially if combined with other biomarkers for mortality [56,57,58,59]. Results from trials and prospective studies are summarized in Table 3.

### 2.4. Cardiovascular, Lung Biomarker, and New Perspectives in COVID-19

Interestingly, the involvement of endothelium has been demonstrated also in myocardial tissue, so Huang et al. used troponin as a cardiac injury biomarker [60]. These results are in accordance with Liaqat et al.’s description of laboratory exams, imaging, and ECG alterations associated with cardiac injury in COVID-19 patients [61]. Ileri et al. investigated the possible role of risk stratification of severe COVID-19 according to troponin levels; furthermore, they investigated the occurrence and severity of thorax CT lesions, in particular the density and volume of pulmonary consolidations, ground glass opacities, and the radiological progression of these lesions [62]. According to Perez et al., lung injury could be assessed and evaluated using the vascular endothelial cadherin biomarker, as it has been demonstrated to have an elevated expression on lung endothelial cells. In fact, alveolar damage and thrombi are the most common lung histopathological lesions reported in patients with severe COVID-19 [63]. Gelzo et al. proposed to monitor metelloproteinases 3 and 9 to monitor and assess the lung damage in COVID-19, as these proteins are involved in lung damage and regeneration [64]. Different and new perspectives have been investigated: Danlos et al. studied the role of metabolomics, which represents post-genomic changes in biochemical circuitries influenced by COVID-19 infection and its treatment. They noticed a major increase in different amino acids, lipids, sugars, and polyamines in severe patients. The exception is represented by tryptophan, whose levels tend to be lower in patients with severe forms of the infection, suggesting a disease-associated activation of its consumption [65]. Furthermore, RAGE, IL-33, the ACE2 receptor, or ST2, which are supported by trial experience, are not easy to measure in everyday clinical practice. This last point should be considered during the clinical monitoring of COVID-19 biomarkers: Il-6, D-dimer, PCT, and CRP are very common in clinical practice, so even if combined, they could be helpful for the patient’s prognosis [2,12] Moreover, Wallentin et al. studied the role of the receptor used by the virus to enter target cells, ACE2. First, they demonstrated higher levels of this marker in elderly male patients with cardiovascular diseases and diabetes, the category of patients with higher risks of developing complications with SARS-CoV-2 infection. From this observation, they hypothesized a direct correlation between ACE2 levels and the risk of severe complications from the infection [11]. Another important biomarker could be RAGE, which is the receptor for advanced glycation end-products expressed by type I pneumocytes. In the past, several studies have underlined the role of this protein in the characterization of lung injury in ARDS. Wick et al. demonstrated a similar role in COVID-19-associated pneumonia [66]. They noticed higher levels of RAGE in patients who required more oxygen support and in those with a worse 5-day outcome. For these reasons, they hypothesized the role of RAGE as a biomarker for identifying patients with probable long-term adverse outcomes. Finally, Zeng et al. also demonstrated the prognostic role of suppression of tumorigenicity-2 (ST2), a member of the toll-like receptor family whose main ligand is Interleukin-33 (IL-33) [67]. They demonstrated higher levels of this biomarker in patients with more severe forms of the infection. Results from trials and prospective studies are summarized in Table 4.

## 3. Limitations

The paper follows the SANRA methodology (Appendix A). The research does not consider all retrospective studies, only the most significant, according to the authors’ opinion. It is important to underline that not all laboratories have the possibility to measure all the biomarkers suggested, especially the endothelial biomarkers, which have a lot of potential but are still expensive and not always available.

## 4. Conclusions

This paper aims to provide quick tools and a compendium for intensive care physicians to perform a quick assessment in cases of severe COVID-19 infections. The article summarizes the main biomarkers from prospective studies and clinical trials: not all of these are available in all hospitals, but a wide knowledge of all possibilities to measure and monitor offers more instruments to the physicians. Only through the early identification of the most serious patients is it possible to provide them with the most appropriate treatments; this point is reached through the continuous search for ideal and functional biomarkers. Il-6, D-dimer, VWF, CRP, and PCT seem to be good COVID-19 biomarkers that are easy to perform in clinical practice. However, other different biomarkers have shown interesting potential and should be included in daily clinical practice.

## Figures and Tables

**Table 1:** It resumes the principal prognostic cytokines in severe COVID-19.

Authors/Year	Type of Study	Biomarker	Patients (Sample)	Outcome
Jøntvedt Jørgensen et al. [18] Year 2020	Prospective studies (Classified also as Trial on Pubmed)	Il-6 and MCP	34 patients	IL-6 and MCP-1 were inversely correlated with P/F
Pirabe et al. [19] Year 2021	Prospective studies (Classified also as Trial on Pubmed)	IL-6, IL-8 and tumor necrosis factor (TNF)	110 patients	Adverse outcomes in elderly are associated with an inappropriate immune response,
Santa Cruz et al. [20] Year 2021	Prospective studies (Classified also as Trial on Pubmed)	Il-6	46 Patients	IL-6 level was the most significant predictor of the non-survivors group,
Espindola et al. [21] Year 2021	Prospective studies (Classified also as Trial on Pubmed)	Il-6 in cerebrum spinal fluid (CSF)	48 patients	Neurological syndromes related to SARS-CoV-2 were associated with high CSF levels of IL-6
Van singer et al. [22] Year 2020	Prospective studies (Classified also as Trial on Pubmed)	Il-6 and endothelial dysfunction biomarkers And TREM-1 myeloid receptor	76 patients	IL-6 measured at presentation to the ED had the best accuracy for 30-day oxygen requirement
Popadic et al. [23] Year 2021	Prospectives studies	Serum albumin, D-dimer, and IL-6	160 patients	Serum albumin, D-dimer, and IL-6 at admission to ICU were independently associated with mortality
Galván-Román et al. [24] Year 2021	Prospectives studies	Il-6 and Tocilizumab response	146 patients	IL-6 greater than 30 pg/mL predicts IMV requirement and it helps in tocilizumab choice
Gordon et al. [25] Year 2021	Clinical Trials	Il-6 and Tocilizumab response	353 patients	Il-6 reduction is associated with tocilizumab response and outcome
Salama et al. [26] Year 2021	Clinical Trials	Il-6 and Tocilizumab response	389 patients	Il-6 reduction is associated with tocilizumab response and outcome
Schultheiß et al. [27] Year 2022	Prospectives studies	Il-6 and long term sequelae	318 patients	Il-6 monitoring is useful for long term sequelae
Queiroz et al. [28] Year 2022	Prospectives studies	Il-6 and long term complications	317 patients	Il-6 monitoring is useful for long term complications
Melero et al. [29] Year 2022	Prospectives studies	IL-8 messenger RNA (mRNA)	Lung Biopsy from 16 patients	Il-8 is associated to nflammatory infiltrates and neutrophil extracellular traps
Bain et al. [30] Year 2021	Prospectives studies	Il-6, Il-8, and Il-10	92 patients	Conclusions: COVID-19 ARDS bears several similarities to viral ARDS
Guasp et al. [31] Year 2022	Prospectives studies	Il-6, Il-8, and Il-10 IL-10, Il-1RA, IP-10	60 patients	levels of pro-inflammatory cytokines do not predict the long-term functional outcome
Han et al. [32] Year 2020	Prospectives studies	Il-6 and Il-10	102 patients	IL-6 and IL-10 can be used as predictors for patients with higher risk of disease deterioration.

**Table 2:** It resumes endothelial and coagulation biomarkers in COVID-19.

Authors	Type of Study	Biomarker	Patients	Outcome
Goshua et al. [33] Year 2020	Prospective studies (Classified also as Trial on Pubmed)	Endothelial biomarker: P-selectin, Von Willebrand factor (VWF) sCD40L, thrombomodulin	68 patients	Endotheliopathy is present in COVID-19 and is likely to be associated with critical illness and death
Vieceli Dalla Sega et al. [34] Year 2021	Prospective studies (Classified also as Trial on Pubmed)	VCAM-1, endothelin-1 and thrombomodulin	54 patients	Endothelin-1 remained stable in nonsurvivors but increased over time in survivors
Al-Samkari et al. [35] Year 2020	Prospective studies (Classified also as Trial on Pubmed)	D-Dimer	400 patients	Elevated D-dimer at initial presentation was predictive of coagulation-associated complications
Nossent et al. [36] Year 2021	Prospective studies	D-dimer and thrombin-antithrombin complexes, in bronchoalveolar lavage fluid	17 patients	Critically ill, with COVID-19 show strong complement system, cytokines, chemokines and growth factors in the bronchoalveolar compartment
Hamzeh-Cognasse et al. [37] Year 2021	Prospective studies (Classified also as Trial on Pubmed)	sCD40L and sCD62P	55 patients	there is a platelet signature of inflammatory response to SARS-CoV-2 infection which varies overtime
Price et al. [38] Year 2022	Prospective studies	Angipoietin 2 (ANGPT2)	102 Patients	COVID-19 ARDS lung autopsy confirmed a link between vascular injury (ANGPT2) and platelet-rich microthrombi
Villa et al. [39] Year 2022	Prospective studies	Angipoietin 2	187 patients	Angiopoietin-2 may be an early and useful predictor of COVID-19 clinical course
Pine et al. [40] Year 2022	Prospective studies	angiopoietin-2, follistatin, and plasminogen activator inhibitor-1 (PAI-1)	49 patients	Elevated markers of endothelial injury were strongly predictive of in-hospital mortality
Smadja et al. [42] Yaer 2020	Prospective studies	Angipoietin 2	40 Patients	Angiopoietin-2 is a relevant predictive factor for ICU direct admission in COVID-19 patients.
Al Otair et al. [47] Year 2021	Prospective studies	Protein C, protein S, antithrombin (AT) III, clotting factor (F) VIII, von Willebrand factor (vWF) and coagulation screening tests (PT and a PTT), fibrinogen, D-dimer	68 patients	The level of vWF is increased early in the course of COVID-19 infection. This can be used as a biomarker for endothelial injury.

**Table 3:** It resumes biomarkers of sepsis in COVID-19.

Authors	Type of Study	Biomarker	Patients	Outcome
Venet et al. [48] Year 2021	Prospective studies (Classified also as Trial on Pubmed)	Plasma IFNα2 levels and IFN-stimulated genes	64 patients	ARDS in SARS-CoV-2 infection appears to be associated with the intensity of immune alterations upon ICU admission
Mellhammar et al. [49] Year 2021	Prospective studies (Classified also as Trial on Pubmed)	Neutrophil-derived heparin binding protein (HBP;	35 patients	HBP is elevated prior to onset of organ dysfunction in patients with severe COVID-19
Smilowitz et al. [52] Year 2021	Prospective studies (Classified also as Trial on Pubmed)	C reactive protein (CRP)	2872 patients	CRP is strongly associated critical illness, and mortality in COVID-19.
Van Singer et al. [57] Year 2022	Prospective studies	Pancreatic Stone Protein	107 patients	CRB-65, CRP and PSP have an excellent accuracy to rule out early mortality in COVID-19.
Lagadinou et al. [58] Year 2022	Prospective studies	Pancreatic Stone Protein	55 patients	The optimal cut-off value to predict prolonged hospital stay was 51 ng/dL
Melegari et al. [59] Year 2023	Prospective studies	Pancreatic Stone Protein	21 Patients	Monitoring PSP plasma levels could be useful in the absence of a specific COVID-19

**Table 4:** Summary of biomarkers of cardiovascular disease, lung biomarkers, and new perspectives in COVID-19.

Authors	Type of Study	Biomarker	Patients	Outcome
Huang et al. [60] Year 2020	Prospective studies (Classified also as Trial on Pubmed)	Troponin and Lymphocyte count	60 patients	The higher levels of troponin T and lower lymphocyte count were predictors of disease progression.
Liaqat et al. [61] Year 2021	Prospective studies (Classified also as Trial on Pubmed)	Troponin and Lymphocyte count	201 patients	COVID-19 disease favors cardiovascular injury among critical and non-critical patients.
Ileri et al. [62] Year 2021	Propsective study	Troponin	74 patients	COVID-19 patients with severe CT findings and progressive disease had higher hs-cTnI levels
Perez et al. [63] Year 2021	Propsective study	CD31, CD34 and vascular endothelial cadherin. Platelet-derived growth factor receptor-β	16 patients (lung biopsy)	These vascular alterations may contribute to the severe and refractory hypoxaemia in COVID-19
Gelzo et al. [64] Year 2022	Prospective studies (Classified also as Trial on Pubmed)	Matrix metalloproteinases (MMP) 3 and 9	108 patients	MMP3 may help to early predict the severity of COVID-19
Danlos et al. [65]	Prospective studies (Classified also as Trial on Pubmed)	Metabolome	72 patients	Metabolome are associated with COVID-19 severity of disease and possible target
Wick et al. [66]	Prospective studies (Classified also as Trial on Pubmed)	RAGE	277 patients	Plasma sRAGE may be a promising biomarker for COVID-19 prognostication
Zeng et al. [67]	Prospective studies (Classified also as Trial on Pubmed)	Serum sST2	80 patients	Serum sST2 levels in nonsurviving cases were persistently high in COVID-19 patients

## Data Availability

Not applicable.

## Appendix A

Scale for the Assessment of Narrative Review Articles: (SANRA)

(1) Justification of the article’s importance for the readership. This review aims to summarize, for intensive care physicians, the current state of knowledge regarding known biomarkers for COVID-19 infection to identify predictors of the most critically ill patients.
(2) Statement of concrete aims or formulation of questions. The aim of the review is to determine the main markers associated with the most severe forms of SARS-CoV-2 infection, to identify those patients at higher risk of death in the early stages of the infection.
(3) Description of the literature search. We searched on PubMed^®^ for the past three years, using the Boolean operators AND, OR, and NOT. We identified all results on the PubMed^®^ database of all studies regarding COVID-19 biomarkers. We selected studies using Boolean operators’ endothelium, cytokines, bacterial infection, and the coagulation biomarker.
(4) Referencing. The review considers and takes into consideration some of the most valuable articles in the field.
(5) Scientific reasoning. To collect the data that we needed to build the article, we specifically used clinical trials regarding COVID-19 biomarkers.
(6) Appropriate presentation of the data. In according to pathophysiological determinants of COVID-19 severity infection, we divided the results into four essential paragraphs: “Cytokine storm”, “Endothelium dysfunction and coagulation biomarkers in COVID-19”, “Biomarker of sepsis”, and “New perspective”.
